# The Arthroscopic Application of Radiofrequency in Treatment of Articular Cartilage Lesions

**DOI:** 10.3389/fbioe.2021.822286

**Published:** 2022-01-20

**Authors:** Chaosheng Lin, Zhenhan Deng, Jianyi Xiong, Wei Lu, Kang Chen, Yizi Zheng, Weimin Zhu

**Affiliations:** ^1^ Department of Sports Medicine, The First Affiliated Hospital of Shenzhen University, Shenzhen Second People’s Hospital, Shenzhen, China; ^2^ Clinical Medical College, Anhui Medical University, Hefei, China; ^3^ Guangdong Key Laboratory of Tissue Engineering, The First Affiliated Hospital of Shenzhen University, Shenzhen Second People’s Hospital, Shenzhen, China; ^4^ Clinical Medical College, Guangxi University of Chinese Medicine, Nanning, China

**Keywords:** cartilage lesions, radiofrequency, articular cartilage, thermal energy, chondroplasty

## Abstract

Articular cartilage lesion is a common disease to be treated by arthroscopic surgery. It will eventually progress to osteoarthritis without proper management, which can affect patients’ work and daily life seriously. Although mechanical debridement and laser have been used clinically for its treatment, due to their respective drawbacks, radiofrequency has drawn increasing attention from clinicians as a new technique with more advantages. However, the safety and efficacy of radiofrequency have also been questioned. In this article, the scope of application of radiofrequency was reviewed following an introduction of its development history and mechanism, and the methods to ensure the safety and effectiveness of radiofrequency through power and temperature control were summarized.

## Introduction

Articular cartilage (AC), a kind of hyaline cartilage covering the articular surface, is a viscoelastic connective tissue composed of chondrocytes and the extracellular matrix (ECM), while ECM is mainly composed of collagen, aggregated proteoglycan and water (Maly et al., 2021). In normal cartilage, the interaction between water, collagen and proteoglycan-rich matrix constitutes the compressive properties and tensile strength of AC. In addition, AC also has excellent wear resistance and lubrication ability (Dutcheshen et al., 2012; Deng et al., 2018).

While participation in sports and daily physical activities, acute impact or chronic injury accumulated by repeated impact of the joint may damage the articular surface and cause AC lesions (Zhang et al., 2019). For example, an earlier study reported full-thickness focal cartilage defects in more than 1/3 of 931 athletes (Flanigan et al., 2010). The cartilage defects play a vital role in cartilage degeneration. Without effective treatment, such degeneration will eventually lead to osteoarthritis (OA) that can affect the patients’ work and daily life seriously and represent a significant health and economic burden on society (Mahmoudian et al., 2021). In a recent epidemiological study, OA occurred in 54.4 million (22.7%) adults, and 43.5% of the patients reported negative impact on their life due to limitation of motion (Barbour et al., 2017). Unfortunately, cartilage has a limited self-repair ability, due to lack of blood vessels, nerves, and lymph tissue. Therefore, the treatment of articular cartilage lesions is still a great challenge in clinical practice (Buckwalter and Mankin, 1998; Ganguly et al., 2010; Huang et al., 2021; Zhou et al., 2021).

Chondroplasty is an effective surgical option for the treatment of AC lesions (Barber and Iwasko, 2006; Gowd et al., 2019). This technique debrides the fibroblast cartilage in the joint to form a smooth and stable articular surface to avoid further degeneration caused by delamination, fragmentation and fibrillation of the injured AC. Chondroplasty includes mechanical debridement, laser, and radiofrequency chondroplasty (Khan and Dillingham, 2002; Rocco et al., 2016). Traditionally, mechanical debridement has been used to debride and smooth the damaged articular surface, but this method usually removes adjacent normal cartilage while treating focal lesions. In addition, it cannot completely smooth the surface of cartilage, which will cause fibrillation on the cartilage surface, leading to further degeneration (Turner et al., 1998; Spahn et al., 2016). As a thermal energy technology, laser has also been applied to treat AC lesions, but it has raised a great concern from clinicians due to safety and cost considerations (Shellock and Shields, 2000; Wienecke and Lobenhoffer, 2003; Cook et al., 2004; Ganguly et al., 2010). In view of the respective deficiencies of the two techniques aforementioned, radiofrequency chondroplasty has recently become a new hotspot as a safer and more effective option (Khan and Dillingham, 2002). In this article, the development history, parameter control, biological research, clinical research, indications, and complications of radiofrequency were comprehensively reviewed.

## Manuscript Formatting

### History of Radiofrequency

#### The Origin and Development of Radiofrequency

In 1891, d’Arsonval et al. reported that radiofrequency waves could increase the temperature of local tissue when passed through, making this thermal energy feasible to be used for clinical application (D'Arsonval, 1891). At the beginning of the 20th century, Cushing et al. used the bovie electric knife to assist in intra-cranial tumor resection, which was a technique using the thermal energy generated by radiofrequency waves to stanch bleeding and remove abnormal tissues (Cushing and Bovie, 1928). In 1975, radiofrequency was applied to treat chronic pain in unilateral limbs (Pawl, 1975). Subsequently, it has been widely used in neurology, oncology, cardiology and other fields (Pawl, 1975; Dickson and Calderwood, 1980; Huang et al., 1987).

#### Application of Radiofrequency in Orthopedic Arthroscopy

Due to the numerous side effects of laser chondroplasty, there was an urgent need to find an alternative method for the treatment of AC lesions (Whipple et al., 1985; Miller et al., 1989; Lane et al., 1997). The application of radiofrequency under arthroscopy played a significant role in the development of orthopedic surgery. In 1986, Schosheim et al. applied radiofrequency under arthroscopy for the first time to perform meniscectomy on rabbit knee joints (Schosheim and Caspari, 1986). The authors found that radiofrequency treatment caused less damage to soft tissues. Turner et al. compared 6 histological variables of the cartilage treated by mechanical debridement and radiofrequency, and reported that the radiofrequency group achieved more favorable results in all the variables, indicating a superior performance of radiofrequency to mechanical debridement for AC lesions (Turner et al., 1998). Since then, an increasing number of studies have supported that radiofrequency is an effective treatment for AC lesions (Allen et al., 2006; Uthamanthil et al., 2006; Edwards et al., 2007). In recent years, there has been a proliferation of relevant clinical studies. However, while gaining more experience, there are still many problems and controversies to be addressed.

### Mechanism of Radiofrequency

#### Types and Grades of Cartilage Lesions

Acute impact or chronic injury of the joint may both damage the articular surface, cause cartilage degeneration, and lead to biomechanical and histological changes of the cartilage, such as joint pain, dysfunction and joint effusion (Buckwalter, 2002). Cartilage lesions can be divided into three types depending on the depth of the damage, namely, partial-thickness defects, full-thickness defects and osteochondral defects. In partial- or full-thickness defects, the damage is completely confined to the cartilaginous tissue and does not penetrate the subchondral bone (Nukavarapu and Dorcemus, 2013). A commonly-used method for classifying cartilage lesions was described by Outerbridge et al. (Slattery and Kweon, 2018). According to the classification of cartilage lesions observed under arthroscopy by the improved Outerbridge system, Grade Ⅰ lesions refer to softened cartilage surface only, which usually does not need to be treated; Grade Ⅱ or Ⅲ lesions refer to a partial-thickness defect with fissures of the cartilage with a diameter less than (Ⅱ) or more than (Ⅲ) 0.5 inches in diameter, which can be treated by mechanical debridement and radiofrequency; Grade Ⅳ lesions refer to subchondral bone exposure, which cannot be effectively treated by radiofrequency, mechanical debridement and other interventions, and needs to take further measures, such as autologous chondrocyte implantation, osteochondral allograft transplantation and so on (Osti et al., 2010). However, the Outerbridge grading system does not take into account the depth of the lesion. On the contrary, the classification system of the International Cartilage Repair Society (ICRS) mainly focuses on the lesion depth (Brittberg and Winalski, 2003). The ICRS Grade Ⅰ lesions are only superficial, such as soft indentation or superficial fissures and cracks. The ICRS Grade Ⅱ lesions have extended to less than half of the cartilage depth, while the ICRS Grade Ⅲ lesions have extended to half or more of the cartilage depth but not yet into the subchondral bone. The ICRS Grade Ⅳ lesions are osteochondral lesions. In addition, some other classification systems focus on histopathology, such as the Osteoarthritis Research Society International (OARSI) system (Pritzker et al., 2006). The OARSI system was classified as 1) uneven but intact chondral surface (OARSI Grade Ⅰ), 2) surface discontinuity (OARSI Grade Ⅱ), 3) vertical fissures (OARSI Grade Ⅲ), 4) erosion (OARSI Grade Ⅳ), 5) denudation of the cartilage (OARSI Grade Ⅴ), or 6) deformation and osteophytes formation of the joint (OARSI Grade Ⅵ).

#### Biomechanical and Histological Changes

Radiofrequency can debride the damaged cartilage surface to create a smooth cartilage surface and improve the mechanical integrity and function of the treated cartilage (Dutcheshen et al., 2012). Through biomechanical modification of the cartilage, the function of the joint can be restored, the degeneration process can be delayed, and the patients’ function and quality of life can be improved. Cook et al. demonstrated that no significant difference in compression stiffness between radiofrequency-treated and untreated AC (Cook et al., 2004). Thus, they believed that radiofrequency had no adverse effect on the biomechanical properties of AC. However, it was obviously not sufficient to judge the biomechanical properties of cartilage only by testing its stiffness. On the other hand, due to the local high temperature caused by the probe and the cartilage defects in the treatment area, the use of radiofrequency might cause secondary damage to the border of the chondromalacic and non-chondromalacic area (Cook et al., 2004). The cartilage defects in the treated area made it impossible to contribute to the bearing capacity of nearby areas. The heat energy would induce the denaturation of collagen, make the collagen shrink, and reduce the immediate and short-term stiffness (Lopez et al., 1998; Edwards et al., 2002a; Uthamanthil et al., 2006). Based on a fiber-reinforced biphasic cartilage model, Dutcheshen et al. inferred that the collagen fibril modulus of the treated cartilage decreased, which might reduce the instantaneous or short-term stiffness (resistance to shear and tension) of AC while increasing the matrix modulus (Dutcheshen et al., 2012). This was beneficial to the restoration of the long-term stiffness (load-bearing capacity) of cartilage.

**FIGURE 1:**
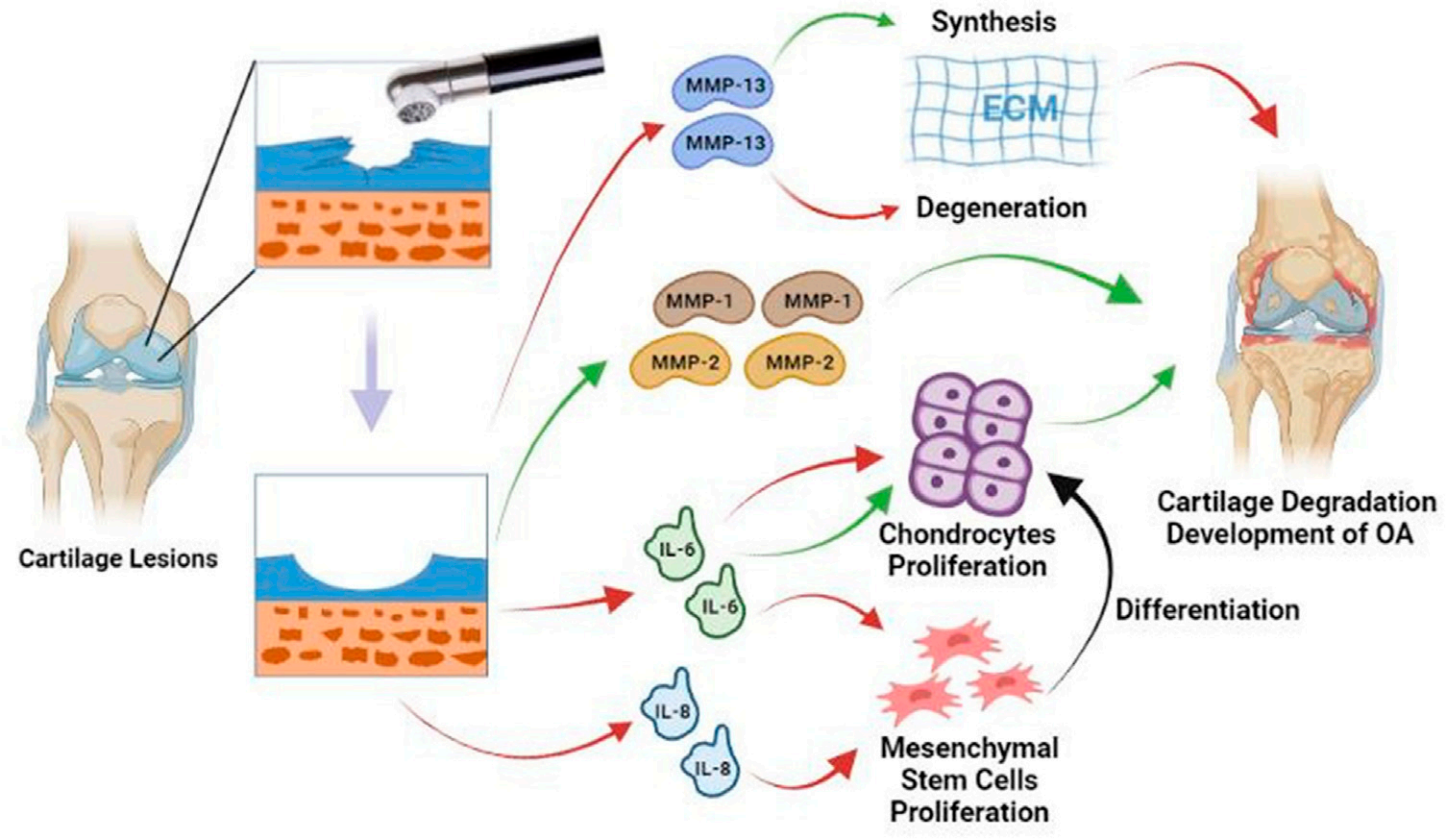
Radiofrequency debrides the damaged cartilage surface to create a smooth cartilage surface. After treatment, MMP-13 tissue immunoreactivity increases, which may lead to a negative shift in the balance between synthesis and degradation of the ECM. IL-6 plays a role in promoting and inhibiting the chondrocyte proliferation. IL-6 and IL-8 promote the proliferation of mesenchymal stem cell. Mesenchymal stem cells can differentiate into chondrocytes and enhance cartilage repair. Secretion of MMP-1 and MMP-2 is significantly reduced, which may help prevent further degradation. Red lines = Stimulating; Green lines = Suppressing; Black line = Differentiation.

Laboratory evidence showed that the use of radiofrequency chondroplasty could not only improve mechanical stability but also reduce the release of inflammatory mediators, which might benefit from the reduction of permeability of the cartilage due to the annealing effect of bipolar radiofrequency energy (bRFE) (Uthamanthil et al., 2006; Dutcheshen et al., 2012). Although the decrease in permeability had a potentially positive effect (i.e., it reduced the release of inflammatory mediators), it affected the exchange of nutrients at the same time. Cook et al. pointed out that the matrix metalloproteinase (MMP)-13 immunoreactivity increased after the bRFE therapy, and the long-term increase of the MMP-13 activity in ECM might lead to a negative balance between ECM synthesis and degradation, which would further accelerate the progression of OA (Cook et al., 2004). MMP-1 can cleave native triple-helical collagens at a single bond, whereas MMP-2 plays a non-specific role in the degradation of fibrillar collagens, basement membrane components, and matrix molecules such as fibronectin. Yasura et al. observed that the secretion of MMP-1 and MMP-2 in AC decreased after the use of monopolar radiofrequency energy (mRFE), which was helpful in preventing the further degeneration of AC (Yasura et al., 2006). Enochson et al. revealed that the expressions of interleukin (IL)-6 and IL-8 in the cartilage were up-regulated after the radiofrequency treatment (Enochson et al., 2012). IL-6 played a role in promoting and inhibiting the chondrocyte proliferation. In addition, it had been proven that IL-6 and IL-8 contributed to proliferation of mesenchymal stem cell which would differentiate into chondrocytes (Figure 1).

#### Monopolar and Bipolar Radiofrequency Energy

**FIGURE 2:**
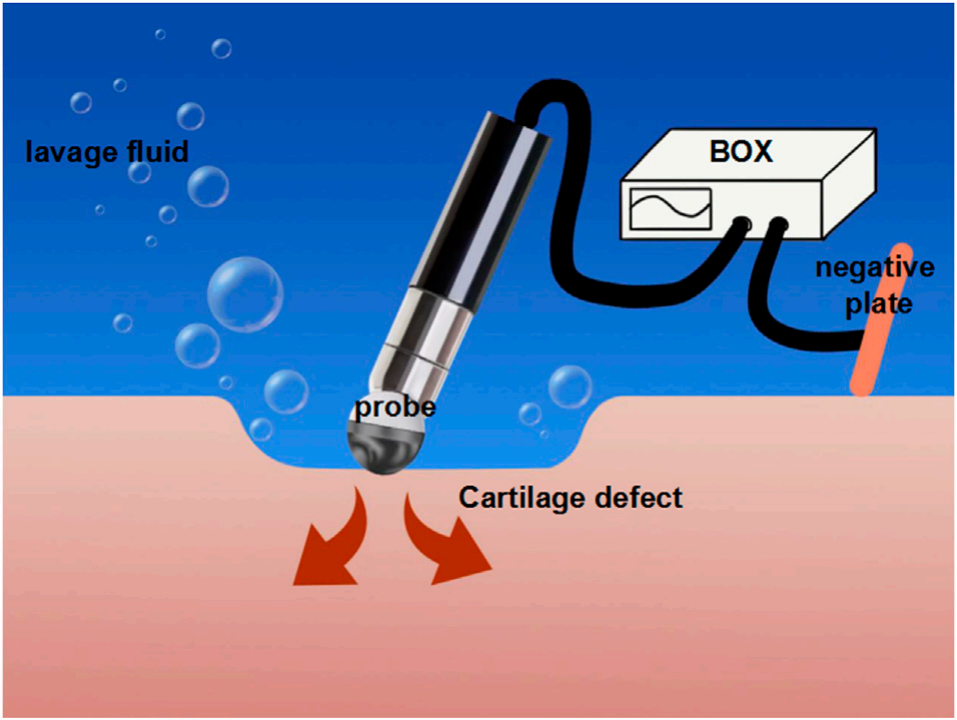
The mechanism of monopolar radiofrequency energy (mRFE).

**FIGURE 3:**
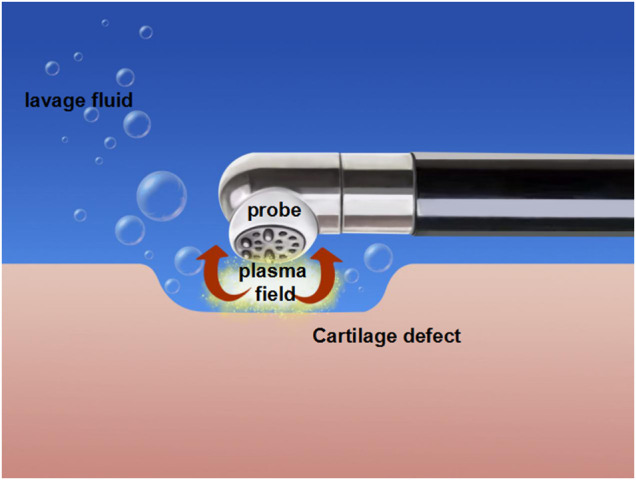
The mechanism of bipolar radiofrequency energy (bRFE).

Radiofrequency equipment includes mRFE (Figure 2) and bRFE (Figure 3), which differ in work mode and temperature distribution (Khan and Dillingham, 2002; Uribe, 2002).

In mRFE, the current from the box reaches the target tissue through the probe in contact with the tissue and then is derived from the return negative plate connected to the patient. Subsequently, the current goes back to the box to form a complete circuit. Because the target tissue has a higher resistance than the rest of the circuit, heat is generated in the target tissue. The system requires the probe to be in contact with the target tissue during the process of treatment, and the energy is directly transmitted to the target tissue, thereby causing an effect similar to electrical damage (Khan and Dillingham, 2002; Ganguly et al., 2010).

Compared with the working mode of mRFE that generates heat, the mode of bRFE is ablation (Good et al., 2009). The bRFE plasma system is a low-temperature ablation technique, which does not directly act on the tissue, but applies a voltage to the conductive fluid between the electrode and the target tissue to generate a plasma field (Wienecke and Lobenhoffer, 2003; Voloshin et al., 2007). Through the impact of charged particles in the plasma field with the target tissue, the molecular chain of the target tissue is broken, thus playing the role of tissue cutting and removal.

The two types of radiofrequency differ greatly not only in work mode, but also in treatment results. Lu et al. reported that significant chondrocyte death occurred after treatment with both mRFE and bRFE, and the depth of death was higher in the bRFE group than in the mRFE group (Lu et al., 2001; Lu et al., 2002a). Edwards et al. explored the temperature distribution of mRFE and bRFE in different depths of cartilage matrix, and the results showed that bRFE led to a higher temperature and more significant chondrocyte death than mRFE (Edwards et al., 2002a; Edwards et al., 2002b). Caffey et al. studied 5 commercially available radiofrequency instruments in human cartilage under simulated surgical conditions, and the results showed that both mRFE and bRFE caused significant cell death in the cartilage under minimal setting conditions (Caffey et al., 2005).

Many studies have suggested that radiofrequency can lead to remarkable chondrocyte death, especially in bRFE, relative to mRFE. However, the cartilage surface becomes smooth after receiving radiofrequency treatment, providing AC with resistance to wearing and tearing, which can delay the progress of cartilage degeneration. Meanwhile, cartilage has a certain self-repair capability (Caplan et al., 1997). Therefore, whether radiofrequency is suitable for treating cartilage lesions in terms of safety and practicability still requires further *in vivo* experiments to validate.

### Control Factors of Radiofrequency

The effectiveness of the radiofrequency therapy can be affected by several variables, including the choice of treatment device, exposure time, power setting, and temperature control and speed of the lavage fluid flow (Table 1).

**TABLE 1:** Influence of radiofrequency time, power setting, temperature control and lavage fluid on articular cartilage.

Factor	Radiofrequency type	Specimen	Main result	Ref
**Time**
5–40s	mRFE、bRFE	42 fresh human knees	after radiofrequency treatment for 15s, the knee surface began to become smooth	Lu et al. (2002a)
0–50s	bRFE	6 fresh bovine knees	At least 20 s of radiofrequency treatment is needed to smooth the surface of the cartilage	Peng et al. (2020)
B1 = continuous treatment, 1 pass	bRFE	36 fresh tibial plateau of pigs	The cartilage becomes smooth in B2 mode	Huber et al. (2020)
B2 = continuous treatment, 2 passes				
**Power setting**	—	—	—	—
Setting 2 vs setting 7; coagulation vs ablation	bRFE	12 fresh porcine knees	Thermal radiation damage can be reduced by ablation mode at high power setting	Wang et al. (2012)
20W–110W		9 healthy adult bovine patellae	The lowest ablation mode setting (60W) resulted in the minimum depth of chondrocyte	Lotto et al. (2006)
20W、40W、60W	bRFE	Paired patellae from 11 horses	Radiofrequency use above 20W is harmful to chondrocytes	Ryan et al. (2003)
50W vs 110W	mRFE	13 healthy viable adult bovine patellae	Tissue effect of cartilage is minimal under 50W power and 25 μm probe	Mitchell et al. (2006)
**Temperature control**	—	—	—	—
45°C、50°C、55°C	N/R	Specimens of arthritic and nonarthritic femoral articular cartilage	At 50°C, the cartilage recovered to a certain degree of thermal stress 1 week after treatment	Kaplan et al. (2003a)
37–65°C	N/R	318 fullthickness cartilage explants from sheep	The death of chondrocytes increased rapidly when the temperature exceeded 50–55°C	Voss et al. (2006)
Control probe distance	bRFE	N/A	The temperature decreases with increasing distance	Kaplan et al. (2003b)
Control the lavage fluid temperature	mRFE	16 fresh human knees	Thermal chondroplasty with 37°C lavage fluid resulted in less depth of chondrocyte death and produced smoother surfaces	Lu et al. (2002b)
Control the irrigation flow of the lavage fluid	bRFE	6 cadavermens	Avoid temperatures above 50°C by using a high irrigation flow	Ahrens et al. (2018)

N/A not applicable, N/R not reported.

#### Time

Clinicians are prone to premature termination of the treatment due to limited arthroscopic magnification when using radiofrequency to treat cartilage lesions, resulting in a less smooth cartilage surface after treatment. Therefore, it is necessary to accurately control the use time of radiofrequency.

Lu et al. performed mRFE and bRFE treatments on fresh osteochondral sections for 5–40s (Lu et al., 2002a). The electron microscope observation showed that the AC surface became smooth with the color gradually turning to yellow after 15 s of radiofrequency treatment in both groups. Excessive treatment time resulted in a significant increase in the depth of chondrocyte death. Similar result was obtained by Peng et al. (Peng et al., 2020). They noted that the glycosaminoglycan content was negatively correlated with exposure time, suggesting that radiofrequency treatment was also negatively correlated with exposure time and had time-dependent damage to the metabolic chondrocyte vitality. Huber et al. compared different modes of radiofrequency therapy. In order to treat a 1 cm^2^ cartilage defect area, one continuous pattern, at least 17 s, is required to smooth the articular cartilage (Huber et al., 2020). The cartilage surface only appeared to become smooth after two continuous radiofrequency treatments. With this continuous treatment pattern extending for a long time, the chondrocyte death could reach up to 95%.

These results indicate that radiofrequency needs to be used for more than 15 s in order to smooth the AC surface during clinical treatment. On the contrary, the exposure time should be controlled as short as possible to reduce the effect on the chondrocyte vitality. Due to different specimens used and obvious variation in the thickness of cartilage in different studies, it is difficult to reach a consensus on this issue.

#### Power Setting

Wang et al. showed that high-power settings in the ablation mode could reduce the thermal radiation injury and was therefore more suitable for the treatment of cartilage lesions (Wang et al., 2012). Lotto et al. discovered that a continuous “char-like layer” was observed on the cartilage surface when the power setting was greater than 60W, which significantly reduced the depth of cell death (Lotto et al., 2006). As the power increased, the current increased significantly. A higher electrical current correlated with increased cell death, even though “char-like layer” appeared in all groups with power higher than 60W. Therefore, they concluded that chondrocytes had the minimum death depth when the cartilage surface was treated at 60W. In a study on the safety of radiofrequency chondroplasty, Ryan et al. found that the cell survival rate of the 40W and 60W groups decreased to 81 and 73%, respectively (Ryan et al., 2003). However, the local peak temperature of the articular surface cartilage was lower than 50°C, at which the chondrocyte vitality might be able to restore (Kaplan et al., 2003a; Voss et al., 2006). Mitchell et al. proposed that in addition to power setting, the selection of probe was also an important factor. Compared with the 110W power and 125 μm probe, the 50W power setting and 25 μm probe had the least influence on the tissue (Mitchell et al., 2006).

In general, different types and manufacturers of radiofrequency will lead to different conclusions. In addition, the experiments mentioned above are only *in vitro* studies or zero-time studies after treatment. Long-term *in vivo* studies are expected to simulate the actual clinical conditions in the future. Since all the manufacturers have provided guidelines for the use of radiofrequency, clinicians can follow these guidelines in clinical practice.

#### Temperature Control

It has been reported in previous studies that radiofrequency chondroplasty can cause AC to be subjected to destructive heat stress, which will further lead to chondrocyte death. Therefore, it is necessary to explore whether there is a temperature “safe zone” when using radiofrequency, at which it will not cause the death of chondrocytes. Kaplan et al. placed cartilage specimens in water baths at 45°C, 50°C and 55°C respectively for up to 3 min and evaluated the chondrocyte vitality immediately and 1 week after treatment (Kaplan et al., 2003a). The chondrocyte vitality was restored 1 week after treatment in the 50°C group, but not in the 55°C group. Voss et al. also confirmed that there was a strong relationship between temperature increase and chondrocyte death (Voss et al., 2006). The chondrocytes were more likely to restore their vitality after thermal injury when the temperature was less than 50°C (Mitchell et al., 2006). Therefore, the local temperature should also be properly controlled to ensure the safety of AC while achieving the desired clinical results.

In another study by Kaplan et al., the temperature was controlled by changing the distance between the bRFE probe and the cartilage surface (Kaplan et al., 2003b). Lu et al. noted that the use of higher temperature lavage fluid could shorten the time required to reach the preset temperature at the beginning of treatment, thus prolonging the effective treatment time (Lu et al., 2002b). In order to prevent the radiofrequency temperature from exceeding 50°C, Ahrens et al. used a higher lavage fluid flow in arthroscopic surgery to reduce the temperature (Ahrens et al., 2018). This method had also been mentioned and confirmed by Good et al. (Good et al., 2009).

In clinical practice, attention should be paid to controlling the local temperature of radiofrequency to prevent the environment of cartilage from exceeding 55°C for a long time. The radiofrequency temperature can be controlled by increasing the lavage fluid flow, shortening the working time of radiofrequency, controlling the distance of the radiofrequency probe, and increasing the initial temperature of the lavage fluid.

### Arthroscopic Application of Radiofrequency

In the arthroscopic application of radiofrequency for the treatment of cartilage lesions, the scope of application and indications of this technique are worthy of attention. The types of patients included in the related clinical studies and the procedures in which the radiofrequency system was used were presented in Table 2.

**TABLE 2:** Indications: Application of radiofrequency in arthroscopy.

Radiofrequency type	Sample size	Subject or therapeutic site	Grade of cartilage lesions	Follow-up time	Main result	Ref
**Knee arthroscopy**
bRFE	25	Patellofemoral joint or tibiofemoral joint	Grade Ⅲ lesions (23/25)	10.4 ± 9.6 months	3 (12%) lesions continued to progress; Defects were partially or completely filled in more than 50% of patients	Voloshin et al. (2007)
bRFE	4	Femoral trochlea, medial condyle, or patella	Grade Ⅲ lesions	N/R	The articular cartilage defects become smooth after radiofrequency treatment	Voloshin et al. (2005)
bRFE	824	Medial femoral condyle, patella and the trochlea	The mean lesion size was 358 mm^2^	129 days	The improvement in the total WOMAC and KOOS scores after treatment with bRFE	Gharaibeh et al. (2018)
mRFE + MD	28	Femoral condyle	Outerbridge Grade Ⅲ lesion 1.5–3.0 cm in diameter	12 and 24 months	Both pain and functional outcomes were significantly improved	Barber and Iwasko, (2006)
mRFE + MD	15	Medial femoral condyles and lateral femoral condyles	7 Grade Ⅱ lesions and 8 Grade Ⅲ lesions	19 months	The score of IKDC was significantly improved	Kang et al. (2008)
**Shoulder arthroscopy**	—	—	—	—	—	—
N/R	88	Patients with PAGCL	N/A	N/R	41 (45%) patients with PAGCL had surgeries involving radiofrequency devices	Solomon et al. (2009)
**Wrist arthroscopy**	—	—	—	—	—	—
mRFE、bRFE	14	14 cadaver arms	N/A	N/A	Peak temperature in the lunate fossa almost reached 70°C even under continuous irrigation	Huber et al. (2015)
(radiofrequency shrinkage)	4	Scapholunate ligament	scapholunate ligament injuries	4.8 years	Patients had significant improvements in pain and satisfaction with outcomes	Jang et al. (2014)
N/R	6	Patients with complications	N/A	N/A	Six cases of complications from use of radiofrequency at wrist arthroscopy were reported	Giddins et al. (2020)
**Hip arthroscopy**	—	—	—	—	—	—
bRFE + microfracture	1	Acetabulum	N/R	4 months	The authors suggest that the cause of chondrolysis in the patient may have been caused by radiofrequency	Más et al. (2015)
N/R	1	Acetabulum	N/R	1 month	The use of radiofrequency during labral excision may have been responsible for the subsequent chondrolysis	Rehan-Ul-Ha et al. (2010)
N/R	3	human hip cadaveric specimens	N/A	N/A	Five-second-interval pulsed lavage is effective in keeping the hip temperature below 50 °C	McCormick et al. (2013)
**Ankle arthroscopy**	—	—	—	—	—	—
bRFE (Ankle debridement)	30	Patients with ankle impingement syndrome	N/A	21.5 months	Meislin, AOFAS and VAS scores were significantly improved compared with preoperative score	Han et al. (2014)
N/R (cystectomy)	7	Symptomatic cystic lesions of the talus	N/A	1 year	The postoperative functional scores of the patients were significantly improved and no complications developed	Zhu et al. (2019)
bRFE	6	Cadaver ankle specimens	N/A	N/A	Use high irrigation flow to avoid temperatures exceeding 50 °C/122°F	Ahrens et al. (2018)

N/A not applicable, N/R not reported.

#### Knee Arthroscopy

Partial-thickness cartilage defects can be detected in more than 60% of the cases that received knee arthroscopy, with Outerbridge Grade Ⅲ lesions of the patella being the dominant one (Curl et al., 1997). Voloshin et al. investigated 15 patients who underwent knee arthroscopic radiofrequency chondroplasty (a total of 25 cartilage lesions, including 11 patellofemoral lesions and 14 tibiofemoral lesions, of which 23 were Outerbridge Grade Ⅲ lesions) (Voloshin et al., 2007). At the second-look, significant reductions in the mean extent of the lesions compared with the preoperative status were observed, and partial or complete filling of the cartilage defects in 56% of the patients and continued progression of cartilage degeneration in only 3 cases (12%) were detected. Similar results were reported in another case report by Voloshin et al. (Voloshin et al., 2005). Gharaibeh et al. retrospectively reviewed 824 patients who underwent bRFE for the treatment of cartilage lesions, where the most common lesion involved was the medial femoral condyle (27%), followed by patella (21%) and pulley (9%), with a lesion area ranging 7–820 mm^2^ (mean 358 mm^2^) (Gharaibeh et al., 2018). Neither postoperative complications nor reoperation in patients were found to be directly related to the use of bRFE. Barber et al. treated 28 patients with Outerbridge Grade Ⅲ lesions of the femoral condyle (1.5–3.0 cm in diameter) using radiofrequency combined with mechanical debridement (Barber and Iwasko, 2006). Similarly, the follow-up study of Kang et al. included patients with Outerbridge Grade Ⅱ (7 cases) or Ⅲ (8 cases) lesions of the medial or lateral condyle of the femur, who were also treated by radiofrequency combined with mechanical debridement (Kang et al., 2008). The clinical results of these patients were significantly improved compared with their preoperative status (Spahn et al., 2016).

Consistently, in several earlier clinical studies, the patients treated by radiofrequency chondroplasty were also classified as Outerbridge Grade Ⅱ or Ⅲ lesions. Most cartilage lesions were on the medial condyle of the femur, followed by lateral condyle, patella and trochlea, while the lesion area was generally less than 800 mm^2^. A majority of the patients included under this standard had achieved good clinical results, with a low incidence of complications. The detailed indication for the use of radiofrequency in knee arthroscopy needs further research and more long-term follow-up studies to confirm.

#### Shoulder Arthroscopy

Radiofrequency has long been used in other arthroscopies as well, and its earliest application was in shoulder arthroscopy to reduce periarticular soft tissue laxity and treat shoulder instability. However, in a review by Solomon et al., it was found that most published studies on the relationship between post-arthroscopic glenohumeral chondrolysis (PAGCL) and surgical factors focused on radiofrequency (Solomon et al., 2009). Around 45% of the patients with PAGCL were treated by radiofrequency during surgery. Hence, the side effects of radiofrequency have discouraged its application in shoulder arthroscopy (McFarland et al., 2002).

#### Hip Arthroscopy

There are few studies on the use of radiofrequency in the treatment of cartilage lesions in hip arthroscopy, and in the relevant studies, radiofrequency was mostly used in iliopsoas release, ligament debridement, treatment for osteoid osteoma and so on (Suarez-Ahedo et al., 2015). In two case reports on the use of radiofrequency to treat acetabular cartilage lesions, both patients developed chondrolysis within 6 months after surgery (Rehan-Ul-Ha et al., 2010; Más et al., 2015). Although there is no direct evidence indicating the cause of chondrolysis by radiofrequency, it has been mentioned in previous reports that the local high temperature produced by radiofrequency led to the death of chondrocytes and even destruction of the entire cartilage layer. Thus, the authors believed that this could be a side effect of radiofrequency.

#### Wrist Arthroscopy

With the rapid development of the radiofrequency technology, small radiofrequency probes have been created to be used in the wrist and ankle joints, mainly for “thermal shrinkage” and “joint capsule shrinkage” treatment of small joint lesions (Huber et al., 2013; Han et al., 2014; Huber et al., 2015; Leclercq and Mathoulin, 2016; Giddins et al., 2020). Jang et al. executed radiofrequency shrinkage in 4 patients with scaphoid ligament injury and achieved significant improvement during a follow-up of 4.8 years (Jang et al., 2014). Zhu et al. reported 7 cases of excision of lesions by radiofrequency in ankle arthroscopy with no complications complained during the follow-up (Zhu et al., 2019).

#### Summary of Radiofrequency Applications in Arthroscopy

Although some laboratory studies had shown that the shoulder temperature could be effectively reduced under the condition of continuous lavage fluid flow, it might still exceed 50°C in a short time interval of limited flow, causing thermal damage to the cartilage (Lu et al., Arthroscopy, 2005, 21, 592–596; Good et al., J Bone Joint Surg Am, 2009, 91, 429–434; Zoric et al., J Bone Joint Surg Am, 2009, 91, 2,448–2,454). For the hip joint, McCormick et al. proposed that 5s-interval pulse irrigation was effective in maintaining the intra-articular temperature below 50°C (McCormick et al., Arthroscopy, 2013, 29, 336–342). For the wrist and ankle joints, the local temperature of cartilage lesions could often exceed 50°C due to a small size and varying cartilage thickness (Huber et al., J Hand Surg Am, 2016, 41, 1,080–1,086). It was also reported that the lavage fluid could not effectively dissipate heat, resulting in PAGCL, chondrolysis, distal radioulnar joint and local skin necrosis (Curtin and Friebe, Orthopedics, 2014, 37, e746–e749). Hence, treatment of cartilage lesions by radiofrequency is rarely seen in other than knee arthroscopy, and the use of “thermal shrinkage” and “joint capsule shrinkage” techniques to ablate the diseased tissue requires particular attention to controlling the local temperature, so as to prevent the occurrence of complications.

### Radiofrequency Compared With Other Treatments

#### Radiofrequency Versus Mechanical Debridement

In 1998, Turner et al. reported that, compared with mechanical debridement, less histological changes and less destruction of cartilage were observed in the treatment with bRFE (Turner et al., 1998). Uthamanthil et al. showed that the postoperative cartilage thickness and stiffness were significantly higher in the mRFE group than in the mechanical debridement group (Uthamanthil et al., 2006). Allen et al. used bRFE and mechanical debridement to treat meniscus injury and cartilage lesions respectively (Allen et al., 2006). The results showed no difference between bRFE and mechanical debridement in terms of the effect on chondrocyte vitality. However, compared with the “tearing” approach of mechanical debridement, radiofrequency could treat cartilage lesions more precisely, create a smoother cartilage surface, and avoid damage to the articular surface.

The earliest prospective clinical study using radiofrequency for the treatment of cartilage lesions was conducted by Owens et al. (Owens et al., 2002). They included 39 patients with Outerbridge Grade Ⅱ or Ⅲ lesions of the patella to compare radiofrequency chondroplasty with mechanical debridement chondroplasty. The patients were evaluated before and after surgery based on the Fulkerson-Shea Patellofemoral Joint Evaluation Score, and the results showed that radiofrequency chondroplasty achieved better clinical outcomes. Clearing the cartilage lesions by radiofrequency cannot regenerate the original tissue, but is effective in relieving symptoms and delaying the progression of cartilage degeneration. This encouraging clinical effect can be explained by the microscopic observations that radiofrequency chondroplasty removes fibrotic cartilage, which is the source of chemical and mechanical irritation in the joint.

Although several studies have reported short-term and medium-term follow-up of radiofrequency treatment of AC lesions, long-term follow-up has not been reported yet. Recently, Spahn et al. published the 1-year, 4-years and 10-years follow-up results of bRFE versus mechanical debridement, respectively (Spahn et al., 2008; Spahn et al., 2010; Spahn et al., 2016). It was found that the subjective Knee injury and Osteoarthritis Outcome Score in the radiofrequency group was better than that in the control group. Although the Tegner score of the two groups was at the same level in the 10-years follow-up results, the radiofrequency group reached a higher level of exercise in the 1- and 4-years follow-up results. The medial joint space narrowed in both groups during the follow-up period, and was narrowing obviously faster in the mechanical debridement group. The results of long-term clinical follow-up showed that radiofrequency chondroplasty achieved a better subjective effect. The level of vitality at the 10-years follow-up was lower than that before operation, which might be explained by the increase in age and the progression of OA. Although neither of the two treatment methods can completely prevent the progression of OA, radiofrequency chondroplasty is able to delay the progression of OA more effectively.

#### Radiofrequency Versus Microfracture

Microfracture has also been used to treat cartilage lesions, relieve knee pain, and restore knee function. Techniques for repairing cartilage injuries by microfracture have been reported in the existing literature (Cerynik et al., 2009). Osti et al. applied radiofrequency and microfracture to treat postoperative Outerbridge Grade Ⅰ-Ⅱ lesions and Outerbridge Grade Ⅲ-Ⅳ lesions, respectively (Osti et al., 2010). At the 2-years and 5-years postoperative follow-up, the findings suggested that microfracture achieved similar results to RF in terms of short-term functional improvement; however, microfracture also failed to prevent the continued progression of OA.

#### Radiofrequency Chondroplasty: A Cost-Effective Technology

Cartilage lesions can eventually progress to OA, which has a enormous impact on the social economy and health (Mahmoudian et al., 2021). Early chondroplasty for cartilage lesions avoids the need for chondrocyte implantation or arthroplasty if the disease progresses further, which potentially decrease the socio-economic burden. In a study on the analysis of the economic benefits of comparing radiofrequency and mechanical debridement, the results showed that radiofrequency chondroplasty resulted in a total cost saving of more than 3000 USD per patient over a 4-years follow-up period. This was attributed to the better efficacy and lower revision rate of radiofrequency (Adeyemi et al., 2020). Moreover, radiofrequency is also considered superior to mechanical debridement in terms of operation time and contributes to the reduction in bleeding, which is facilitated by the coagulation effect of radiofrequency on the small vessels in the adjacent tissue (Camillieri et al., 2001; Cetik et al., 2009). For doctors and patients, this is clearly the treatment of choice.

### Safety and Efficacy

#### Thermal Damage

It has been mentioned above that the use of radiofrequency may expose the surrounding healthy cartilage to heat stress and affect the chondrocyte vitality, but when the temperature is controlled at 50°C, the chondrocyte vitality will be able to restore to a certain extent after 1 week (Kaplan et al., 2003a). In order to avoid the thermal damage caused by radiofrequency, it is necessary to set appropriate working conditions. Firstly, the probe with a chondroprotective design can be used, and the power should be set appropriately according to the manufacturer’s guideline before the surgery (Huang et al., 2014). Secondly, the use time and probe distance must be precisely controlled during the surgery, because the damage of radiofrequency on the cartilage is time-dependent and the increase of probe distance can significantly reduce local temperature (Kaplan et al., 2003b). Last but not least, appropriate initial temperature and sufficient lavage fluid flow are required to ensure that radiofrequency can reach the working temperature more quickly and maintain an appropriate temperature environment in the joint (Lu et al., 2002b; Ahrens et al., 2018).

#### Osteonecrosis

The local high temperature generated by thermal energy equipment will not only affect the chondrocyte vitality, but also cause deeper bone damage. As mentioned earlier, Lu et al. revealed that bRFE penetrated deeper into the cartilage than mRFE, and hence the energy might also penetrate deeper into the subchondral bone and lead to osteonecrosis (Lu et al., 2001). It has been reported that subchondral bone necrosis occurs after the use of laser and radiofrequency thermal energy equipment in arthroscopic meniscectomy (Muscolo et al., 1996; Rozbruch et al., 1996; Encalada and Richmond, 2004; Cetik et al., 2009). Mehmet et al. found that the addition of radiofrequency chondroplasty to meniscectomy did not increase the incidence of osteonecrosis (Türker et al., 2015). Therefore, the occurrence of osteonecrosis following the use of thermal energy equipment during meniscectomy may be attributed to the increase of tibiofemoral contact pressure due to the decrease of weight-bearing area after meniscectomy, which may lead to subchondral bone microfracture and synovial fluid leakage in the bone, thereby resulting in osteonecrosis (Encalada and Richmond, 2004). In comparison, the use of radiofrequency equipment will not cause osteonecrosis. Similarly, what is desired is to guarantee that radiofrequency is used in a relatively safe environment, which can be achieved by taking the same steps as preventing thermal damage to ensure that local temperature does not exceed 50°C.

#### Is Radiofrequency Chondroplasty Really Safe?

After over 10 years of clinical application, accumulated evidence has supported the effectiveness of radiofrequency in the treatment of cartilage injury, which appears to be superior to mechanical debridement. This includes the long-term follow-up study conducted by Spahn et al. for 10 years. Recently, Koller et al. put forward an opposite point of view (Koller et al., 2020). They planned to use the magnetic resonance imaging T2 Mapping technique to quantitatively evaluate the efficacy of radiofrequency chondroplasty in patients with Outerbridge Grade Ⅱ lesions of the patella 1 year after surgery. However, the trial was terminated prematurely because the T2 Mapping quantitative analysis of 5 patients showed postoperative cartilage lesions. Koller et al. believed that radiofrequency was often combined with meniscus repair in treating cartilage lesions, so the improvement in the postoperative functional score might be due to meniscus repair. T2 Mapping is a new and sensitive MR technique, which can provide information about the interaction between extracellular matrix and cartilage water molecules, and better reflect the cartilage recovery of postoperative patients (Banjar et al., 2021). In particular, specific techniques for glycosaminoglycan (GAG) assessment, such as delayed gadolinium-enhanced MRI has also shown utility in detecting AC damage (Link et al., 2017). T2 Mapping has been widely used for the assessment of cartilage activity as a non-invasive technique, and MRI techniques such as delayed gadolinium-enhanced MRI are also promising. Future studies on the efficacy of radiofrequency will need to make greater use of these techniques.

## Future Outlook

Radiofrequency has been proposed as an effective method for the treatment of cartilage lesions. However, since the local high temperature produced by radiofrequency will affect the chondrocyte vitality, it is necessary to control the use time, power setting and temperature adjustment precisely, so as to obtain a sufficiently smooth surface of the cartilage while avoiding the side effects of radiofrequency. In terms of clinical application, although a large number of clinical studies (even including 10-years follow-up studies) have proven the effectiveness and safety of radiofrequency, there is still a lack of follow-up studies to evaluate the cartilage growth at the biochemical level. Therefore, more effort is needed to include other indicators such as T2 Mapping, which can be used to evaluate the status of cartilage *in vivo*, to further confirm the safety of radiofrequency.

## Conclusions

Cartilage lesion is a disease of AC loss caused by acute injury or repeated injury, which will eventually develop into OA without proper management. As an alternative to mechanical debridement and laser for the treatment of cartilage lesions, radiofrequency has achieved encouraging results in the past 2 decades. However, there is insufficient evidence to support its indications and safety. At present, we believe that, when radiofrequency is used to treat cartilage lesions, it is necessary to control its indications, mainly for Outerbridge Grade Ⅱ or Ⅲ lesions of the knee cartilage, and the range of lesions should generally not exceed 800 mm^2^. The use of radiofrequency in such cases seems to be safe with fewer complications.

## Abbreviations

AC: articular cartilage; bRFE: bipolar radiofrequency energy; ECM: extracellular matrix; ICRS: International Cartilage Repair Society; IL: interleukin; MMP: matrix metalloproteinase; mRFE: monopolar radiofrequency energy; OA: osteoarthritis; OARSI: Osteoarthritis Research Society International; PAGCL: post-arthroscopic glenohumeral chondrolysis.
